# Retraction: Knockdown of TRIM65 inhibits lung cancer cell proliferation, migration and invasion: A therapeutic target in human lung cancer

**DOI:** 10.18632/oncotarget.28571

**Published:** 2024-05-16

**Authors:** Xiao-Lin Wang, Wei-Ping Shi, Hong-Can Shi, Shi-Chun Lu, Kang Wang, Chao Sun, Jian-Sheng He, Wei-Guo Jin, Xiao-Xia Lv, Hui Zou, Yu-Sheng Shu

**Affiliations:** ^1^Department of Thoracic Surgery, Northern Jiangsu People’s Hospital and Clinical Medical College of Yangzhou University, Yangzhou 225001, People’s Republic of China

**This article has been retracted:** Multiple instances of image duplication were discovered in this paper. Specifically, there are overlapping images within Figure 7B representing IHC staining of two different conditions. Additionally, the Trim65 blots in Figure 1E are duplicates of those in Figure 1C of an earlier published paper [1], and part of the GAPDH blots, also in Figure 1E, overlaps with those in Figure 5B from a second earlier published paper that has since been retracted [2]. Therefore, with the agreement of all authors, the Scientific Integrity office at Oncotarget has decided to retract this paper.

Original article: Oncotarget. 2016; 7:81527–81540. 81527-81540. https://doi.org/10.18632/oncotarget.13131

## References

[1] Liu F , Zong M , Wen X , Li X , Wang J , Wang Y , Jiang W , Li X , Guo Z , Qi H . Silencing of Histone Deacetylase 9 Expression in Podocytes Attenuates Kidney Injury in Diabetic Nephropathy. Sci Rep. 2016; 6:33676. 10.1038/srep33676. 27633396 PMC5025656

[2] Zhang Q , Chen X , Zhang X , Zhan J , Chen J . Knockdown of TMEM14A expression by RNAi inhibits the proliferation and invasion of human ovarian cancer cells. Biosci Rep. 2016; 36:e00298. 10.1042/BSR20150258. . Retraction in: Biosci Rep. 2023; 43:BSR-2015-0258_RET. 10.1042/BSR20150258. 26896463 PMC4759611

